# Supplementary material for: Polymerized Whey Protein Concentrate-Based Glutathione Delivery System: Physicochemical Characterization, Bioavailability and Sub-Chronic Toxicity Evaluation
Source: Molecules. 2021 Mar 24;26(7):1824. doi: 10.3390/molecules26071824 (PMC8037743; doi:10.3390/molecules26071824)
Supplement: Supplementary file 1 [file molecules-26-01824-s001.pdf]

Table S1 Hematology of rats in 28-day toxicity study

Parameter	males				females			
	0%	0.5%	1%	4%	0%	0.5%	1%	4%
red blood cells (RBC) (10^{12} L)	6.26 ±1.07	6.589 ±0.46	6.63 ±0.20	6.50 ±0.42	6.25 ±0.30	6.53 ±0.35	6.76 ±0.56*	6.61 ±0.28
mean corpuscular volume (MCV) (fL)	63.74 ±2.64	62.85 ±2.45	61.72 ±1.91	61.73 ±1.21	58.75 ±2.07	59.71 ±1.45	59.74 ±2.29	59.26 ±1.70
red cell volume distribution (RDW) %	21.64 ±0.22	21.72 ±0.37	21.73 ±0.42	21.57 ±0.22	20.95 ±0.45	20.90 ±0.38	20.86 ±0.23	21.10 ±0.39
Hematocrit (HCT) %	39.82 ±2.72	41.42 ±3.36	40.93 ±1.11	40.11 ±2.20	36.73 ±1.78	38.96 ±1.60	40.30 ±2.64	39.11 ±1.29
platelet count (PLT) (10^9/L)	1020.33±209.37	1215.60±167.71	1140.17±158.03	1232.43±185.88	1137.75±127.57	1271±130.78	1308±117.74	1252.14±147.11
mean platelet volume (MPV) (fL)	6.96 ±0.27	6.38 ±0.40*	6.55 ±0.29	6.39 ±0.18*	6.75 ±0.73	6.30 ±0.35	6.45 ±0.38	6.46 ±0.48
white blood cells (WBC) (10^9/l)	4.20 ±2.19	4.60 ±1.12	3.42 ±1.07	3.88 ±1.50	3.81 ±2.17	4.64 ±1.34	5.86 ±1.76	4.01 ±0.93
hemoglobin (HGB) (g/dL)	13.78 ±2.14	14.13 ±1.30	14.22 ±0.37	14.09 ±0.77	13.18 ±0.56	13.97 ±0.55	14.46 ±0.88*	14.10 ±0.36*
mean corpuscular hemoglobin (MCH) (pg)	22.10 ±1.11	21.48 ±1.11	21.43 ±0.67	21.71 ±0.47	21.13 ±0.61	21.41 ±0.48	21.48 ±0.73	21.40 ±0.63
mean corpuscular hemoglobin concentration (MCHC) (g/dL)	0.78 ±0.35	0.65 ±0.27	0.21 ±0.09	0.29 ±0.09	35.99 ±0.43	35.87 ±0.38	35.95 ±0.47	36.10 ±0.35
lymphocyte (LYM) (g/dL)	3.44 ±1.76	4.05 ±1.06	2.98 ±0.93	3.39 ±1.32	3.19 ±1.65	3.94 ±1.13	5 ±1.26	3.43 ±0.59
granulocyte (GRAN) (g/dL)	0.62 ±0.43	0.43 ±0.30	0.33 ±0.15	0.38 ±0.23	0.51 ±0.54	0.57 ±0.25	0.70 ±0.53	0.48 ±0.38
monocyte (MONO) (g/dL)	0.14 ±0.05	0.12 ±0.041	0.1 ±0	0.11 ±0.03	0.13 ±0.05	0.14 ±0.07	0.16 ±0.07	0.13 ±0.051
Lymphocyte (LYM)%	83.24 ±4.25	90.76 ±1.96*	87.23 ±1.48	87.62 ±3.62	84.91 ±5.25	84.80 ±5.03	86.20 ±4.43	85.94 ±5.29
Granulocyte (GRA)%	83.24 ±4.25	88.30 ±6.28	87.23 ±1.49	87.62 ±3.62	84.91 ±5.25	84.80 ±5.03	86.20 ±4.43	85.94 ±5.29
percent monocytes (MON)%	1.98 ±0.24	1.47 ±0.58	1.70 ±0.45	1.57 ±0.41	84.91 ±5.25	84.80 ±5.03	86.20 ±4.43	85.94 ±5.29

Note: * means significant level is 0.05, ** means significant level is 0.01 compared with the control group

Table S2 Serum biochemistry of rats in 28-day toxicity study

	**males**				**females**			
Parameter	0%	0.5%	1%	4%	0%	0.5%	1%	4%
albumin (ALB) (g/L)	34.78 ±1.05	33.90 ±1.34	33.53 ±0.99	33.01 ±1.34*	34.91 ±1.28	34.53 ±1.35	36.14 ±1.56	34.04 ±1.11
total protein (TP) (g/L)	66.39 ±4.69	63.52 ±3.90	62.75 ±2.46	59.11 ±7.98	64.32±2.90	63.82±3.95	66.51±3.24	64.47±3.50
globulin (GLOB) (g/L)	32.71 ±3.08	29.62 ±3.13	29.22 ±2.36	28.1 ±2.77*	29.42 ±2.22	29.29 ±3.29	31.88 ±3.47	30.42 ±2.75
albumin/globulin (A/G)	1.12 ±0.14	1.15 ±0.11	1.16 ±0.10	1.18 ±0.10	1.19 ±0.09	1.19 ±0.12	1.15 ±0.13	1.13 ±0.09
total bilirubin (TB) (μmol/L)	< 1.0	< 1.0	< 1.0	< 1.0	< 1.0	< 1.0	< 1.0	< 1.0
aspartate aminotransferase (AST) (U/L)	138.20 ±17.63	105.00 ±11.51*	81.60 ±10.14*	91.17 ±8.13*	87.20 ±10.32	103.16 ±8.70	99.50 ±9.98	134.50 ±5.44
alanine aminotransferase (ALT) (U/L)	45.70 ±5.85	43.89 ±6.31	40.50 ±4.55	39 ±7.73*	40.00 ±4.50	41.62 ±3.54	38.25 ±3.86	40.28 ±3.35
amylase (AMY) (U/L)	2320.37 ±178.97	1898.71 ± 145.35*	2009.2 ±194.24*	1775.0 ±110.55*	1442.25 ±148.93	1464.66 ±147.65	1220.55 ± 125.14*	1191.37 ± 120.09*
creatinine (Crea) (μmol/L)	< 4	< 4	< 4	< 4	< 4	< 4	< 4	< 4
creatine kinase (CK) (U/L)	1110.00 ±92.28	723.00 ±64.50*	354.00 ±32.04**	413.40 ±31.05**	1054.60 ±197.79	411.20 ±43.84**	540.50 ±90.78**	363.00 ±61.57**
triglyceride (TG) (μmol/L)	0.77 ±0.37	0.51 ±0.26	0.56 ±0.17	< 0.3	0.42 ±0.21	0.43 ±0.27	0.38 ±0.17	0.35 ±0.09
glucose (GLU) (μmol/L)	7.92 ±0.95	7.17 ±0.51	7.03 ±1.12	6.203 ±0.52*	8.66 ±0.82	8.16 ±1.48	6.89 ±0.58**	6.40 ±1.02**
calcium (Ca) (μmol/L)	2.57 ±0.05	2.50 ±0.08	2.45 ±0.06**	2.379 ±0.10**	2.49 ±0.09	2.53 ±0.07	2.55 ±0.08	2.51 ±0.11
inorganic phosphorus (IP) (μmol/L)	3.35 ±0.31	3.03 ±0.36	3.25 ±0.48	2.70 ±0.23	2.48 ±0.31	2.98 ±0.37*	2.89 ±0.23*	3.02 ±0.42*
blood urea nitrogen (BUN) (μmol/L)	5.06 ±0.58	4.52 ±0.69	5.13 ±0.72	4.48 ±0.88	5.07 ±0.97	4.91 ±1.19	5.68 ±0.52	5.61 ±1.74

Note: * means significant level is 0.05, ** means significant level is 0.01 compared with the control group.
